# Riboswitch-controlled IL-12 gene therapy reduces hepatocellular cancer in mice

**DOI:** 10.3389/fimmu.2024.1360063

**Published:** 2024-03-15

**Authors:** Matthias J. Düchs, Ramona F. Kratzer, Pablo Vieyra-Garcia, Benjamin Strobel, Tanja Schönberger, Peter Groß, Ghaith Aljayyoussi, Aradhana Gupta, Isabel Lang, Holger Klein, Sandra Martinez Morilla, Stefan Hopf, John Park, Sebastian Kreuz, Matthias Klugmann, Frederik H. Igney

**Affiliations:** ^1^ Research Beyond Borders, Boehringer Ingelheim Pharma GmbH & Co. KG, Biberach an der Riss, Germany; ^2^ Cancer Immunology and Immune Modulation, Boehringer Ingelheim Pharma GmbH & Co. KG, Biberach an der Riss, Germany; ^3^ Cancer Immunology and Immune Modulation, Boehringer Ingelheim RCV GmbH & Co. KG, Vienna, Austria; ^4^ Drug Discovery Sciences, Boehringer Ingelheim Pharma GmbH & Co. KG, Biberach an der Riss, Germany; ^5^ Nonclinical Drug Safety, Boehringer Ingelheim Pharmaceuticals, Inc., Ridgefield, CT, United States; ^6^ Global Computational Biology and Digital Sciences, Boehringer Ingelheim Pharma GmbH & Co. KG, Biberach an der Riss, Germany; ^7^ Cancer Immunology and Immune Modulation, Boehringer Ingelheim RCV GmbH & Co. KG, Ridgefield, CT, United States

**Keywords:** il-12, cancer, immunotherapy, aptazyme riboswitch, inducible gene expression, regulatable gene therapy, AAV, Tet-ON

## Abstract

Hepatocellular carcinoma (HCC) and solid cancers with liver metastases are indications with high unmet medical need. Interleukin-12 (IL-12) is a proinflammatory cytokine with substantial anti-tumor properties, but its therapeutic potential has not been realized due to severe toxicity. Here, we show that orthotopic liver tumors in mice can be treated by targeting hepatocytes via systemic delivery of adeno-associated virus (AAV) vectors carrying the murine IL-12 gene. Controlled cytokine production was achieved *in vivo* by using the tetracycline-inducible K19 riboswitch. AAV-mediated expression of IL-12 led to STAT4 phosphorylation, interferon-γ (IFNγ) production, infiltration of T cells and, ultimately, tumor regression. By detailed analyses of efficacy and tolerability in healthy and tumor-bearing animals, we could define a safe and efficacious vector dose. As a potential clinical candidate, we characterized vectors carrying the human IL-12 (huIL-12) gene. In mice, bioactive human IL-12 was expressed in a vector dose-dependent manner and could be induced by tetracycline, suggesting tissue-specific AAV vectors with riboswitch-controlled expression of highly potent proinflammatory cytokines as an attractive approach for vector-based cancer immunotherapy.

## Introduction

Hepatocellular carcinoma (HCC) is the most common form of liver cancer and the third most common cause of all cancer-related deaths worldwide (1, 2). In patients with colorectal carcinoma (CRC) the liver is the most frequent site of metastasis, and fewer than 20% of patients diagnosed with metastatic CRC survive more than 5 years (3). Despite marked improvements in the management of these diseases, there is still a high unmet medical need, specifically to enable long-term tumor control.

IL-12 is a cytokine with strong immune-stimulating activity and has a key function in the induction and enhancement of cell-mediated immunity (4, 5). It is mainly produced by dendritic cells, monocytes, macrophages and B cells, and is involved in the induction of Th1 cell differentiation, activation of T and NK cells, and reprogramming of immunosuppressive cells. IL-12 is comprised of two subunits, p35 and p40, that are linked by disulfide bonds to form a p70 heterodimer. The IL-12 signaling pathway leads to phosphorylation of the transcription factor STAT4, which induces the proinflammatory and anti-tumor cytokine interferon-γ (IFNγ).

In preclinical studies, robust anti-tumor activity of IL-12 has been observed (4, 5). Clinical trials with systemically administered recombinant human IL-12 have caused severe toxicities, including on-study deaths (4–7). Therefore, several approaches to target the protein to tumors while minimizing systemic exposure are under evaluation (6, 7). Local delivery of the genes encoding IL-12 represents an ideal strategy for achieving sustained intratumoral IL-12 levels and reduction of side-effects (6).

Viral platforms, including adenovirus (Ad) or adeno-associated virus (AAV) vectors, have been used for local and sustained expression of IL-12. Intratumoral delivery is a pre-requisite for the reported efficacy following Ad.IL-12 gene therapy in a variety of preclinical cancer models [reviewed by (6)].

The ecdysone receptor-based RheoSwitch^®^ Therapeutic System (RTS) incorporated in Ad-RTS-hIL-12 has extended survival in a mouse glioma model (7). The RTS gene switch relies on constitutively expressed transcription factors that are activated by oral administration of the synthetic small molecule veledimex. Patients with recurrent high-grade glioma who were treated by injection of this vector into the resection cavity wall and subsequent induction of IL-12 expression presented with inflammatory infiltrates at the tumor site, and indications of survival prolongation were observed (8). Severe adverse events reversed upon withdrawal of veledimex.

Hepatotropic AAVs carrying the IL-12 gene for constitutive expression can be administered systemically and were shown to reduce tumor burden and increase survival time in an orthotopic HCC model, suggesting that transduced hepatocytes produce IL-12 that stimulate anti-tumoral hepatic lymphocytes (9). In a technically more sophisticated approach, a liver-specific tetracycline (tet)-On system (10) was employed for regulatable AAV-mediated IL-12 expression following intravenous administration in a model of CRC (11). Induction of IL-12 transgene expression was achieved by oral doxycycline which prevented the establishment of liver metastases and induced a T-cell memory response to tumor cells without obvious side effects. A common feature of these gene switches is that they utilize protein-based systems that activate transcription upon ligand-binding. Ligand-dependent riboswitches, in contrast, function via mRNA-self-cleavage independently of co-expressed foreign proteins and inherently lack any immunogenicity risk.

The concept of this study was to transduce hepatocytes and have them express IL-12 that leads to activation of T and NK cells that infiltrate and attack tumor cells in the vicinity. We employed the previously described tet-dependent riboswitch K19 (12, 13) that allows potent regulation of AAV-based transgene expression (12) for controlling IL-12 expression in mice bearing orthotopic liver tumors. K19 is a synthetic hammerhead switch previously identified from a rationally designed riboswitch library (13) based on the Cb28 tetracycline aptamer (14) and the full-length hammerhead ribozyme N79 from Schistosoma mansoni (15). AAV8 was used as gene delivery vector due to its established hepatocyte tropism (16, 17). Vectorized IL-12 led to IFNγ production, infiltration of T cells and, ultimately, to tumor regression. We also optimized vectors carrying the human IL-12 gene in healthy mice and provide a pharmacokinetic/pharmacodynamic (PK/PD) model that can be used to predict safe and efficacious doses. In summary, these data demonstrate that riboswitch-enabled IL-12 gene therapy can be controlled in a spatio-temporal manner for a safe and efficient immunomodulatory effect using a rational combination of AAV serotype, vector dose, and tet dosing regimen.

## Materials and methods

### Expression constructs

DNA sequences encoding murine or human single-chain IL-12 were derived from previously described constructs IL-12.p40.L.Λp35 and IL-12.p35.L.Λp40 using the (Gly4Ser)_3_ linker (18). The human IgG signal peptide coding sequence was introduced by PCR and cloned into pCR-TOPOP3.3. Constructs harboring the liver-specific LP1 promoter (19) to express IL-12 or a control anti-FITC scFv antibody (12), were obtained by restriction enzyme-mediated cloning utilizing published plasmid backbones (12) or by full gene synthesis (GeneArt, Thermo Fisher). The K19 riboswitch sequence flanked by (CAAA)_3_ spacers has been described previously [(13)]. AAV-cis plasmids carried expression cassettes with SV40 poly-adenylation (pA) signal and were flanked by AAV2 inverted terminal repeats (ITRs). A set of optimized AAV constructs was designed and synthesized to express huIL-12 or mIL-12 driven by the LP1 promoter with the SV40 intron and a bovine growth hormone (bGH) polyadenylation (pA) signal. For these optimized constructs, coding sequences were modified to deplete CpG motifs in order to reduce the risk of uncontrolled immune response via Toll-like receptor 9 (TLR9) (20). These constructs also included 30-nt sequences (insulators) between the expression cassette and the ITRs in order to limit potential influence of the inherent transcriptional activity of the ITRs (21) The insulators were designed to avoid splice sites or transcription factor binding sites. Separately, the presence of C.G-rich tails immediately outside the ITRs in the plasmid backbone has been shown to exert a destabilizing effect that can lead to diminished AAV producibility (22). To address this, we replaced these sequences with 30-nt sequences according to the insulator design.

### Production of recombinant AAV vectors

Production and quantification of AAVs has been described previously (23). Briefly, AAVs were packaged in transiently transfected HEK293 cells and purified by PEG-precipitation, iodixanol-gradients and ultrafiltration. Genomic titers were determined by ddPCR. The three co-transfected plasmid constructs were as follows: One plasmid encoding the AAV cap gene; The AAV cis-plasmid containing the expression cassette flanked by ITRs; and a pHelper plasmid (AAV Helper-free system, Agilent).

### Animals, treatments, and tumor model

8-10 week-old female C57BL/6NCrl mice used in this study were purchased from Charles River laboratories. AAVs were diluted to the desired concentration in PBS and administered into the tail vein in a volume of 100 µL per mouse, under isoflurane anesthesia (3.5-5 volume %). Tetracycline (tet) was formulated in 2-hydroxypropyl beta-cyclodextrin solution (HP-ß-CD, Sigma Aldrich) and administered as described previously (12). Preparation of tet hydrocholoride solution formulation in 20% Kolliphor HS15 was done as follows: 1M NaOH was added to tet HCl (Sigma Aldrich, CAS No.: 64-75-5) and the mixture was ultrasonicated for eight minutes to yield a light-yellow suspension.

Then, a 40% (w/v) solution of Kolliphor HS15 (Sigma Aldrich, CAS No.: 70142-34-6) and was added and again the formulation was ultrasonicated for eight minutes to yield a yellow solution. The pH was adjusted to 5 with 1M NaOH. Finally, deionized water was added resulting in final concentration of 20% (w/v) Kolliphor HS15 and the solution was sterile filtered.

20 µL of blood was collected by puncturing the *Saphenous vein* using K3-EDTA Microvette POCT 20 µL capillary microtubes (Sarstedt) followed by centrifugation. Blood plasma was used for quantitative measurements of biomarkers and IL-12. At the final blood draw, additional serum samples were prepared for the assessment of liver enzymes. For necropsy, animals were anaesthetized with isoflurane (3.5-5 volume %) and then euthanized via cervical dislocation. Organs of interest were dissected and snap-frozen in liquid nitrogen for DNA extraction or the preparation of protein tissue lysates. For tumor-cell application and spleen resection mice received Meloxicam (1.0 mg/kg in 10.0 ml/kg) subcutaneously 1 - 2 hours before surgery with a repetition 24 hours later. Then mice were anaesthetized with isoflurane (3.5-5 volume %) and luciferase-expressing Hepa1-6 tumor cells (1.0 x 10^6^ cells in 50µL PBS) were injected into the spleen of each mouse and allowed to migrate into the liver for 5 min via splenic vein. Thereafter the spleen was resected. All animal experiments in this study were approved by the local German authorities and conducted in compliance with the German and European Animal Welfare Acts.

### Bioluminescence imaging

Tumor volumes were determined by *in vivo* bioluminescence using an IVIS^®^ Lumina III BLI system (Perkin Elmer) with a CCD-camera. For this purpose, 150 mg/kg (7.5 mL/kg) D-Luciferin in aqua dest. was injected *i.p.* 8 min before anesthetization. Light emission was measured 10 min post injection. Tumor-bearing mice were block-randomized according to tumor sizes measured by the *in vivo* BLI of the same day. For block-randomization, a robust automated random number generation within individual blocks was used (MS-Excel 2016).

### Preparation of protein tissue lysates

Snap-frozen tissue samples were homogenized in 100 µL MSD lysis buffer (R60TX-2), using a Precellys-24 homogenizer (KT03961-1-009.2, VWR). Following addition of 900 µL lysis buffer, a second homogenization was carried out. Samples were then centrifuged. 700 µL of supernatant was recovered and protein concentration was determined using a BCA assay (Promega).

### Determination of IL-12 and inflammatory biomarkers

Expression of IL-12, IL-15, IL-18, CXCL10, TNFα, and IFNγ was analyzed using the Mouse IL-12p70 Kit and the Proinflammatory Panel 1 Mouse Kit (K152ARB and K15048D, MSD) or the Human IL-12p70 Kit (K151QVD, MSD). The lowest standard of provided IL-12p70 was taken as lower limit of detection (LLOD). STAT4 and pSTAT4 were quantified using the respective MSD Kits (K150O and K150P).

### Assessment of AST, ALT and GLDH enzyme activity

All measurements were performed using the Konelab PRIME 60 fully automated clinical chemistry analyzer and test kits from Thermo Scientific (according to the Konelab Chemistry Information Manual 12A/2003, March 2003) and spectrophotometrical assessment at 340 nm. Aspartate aminotransferase (AST) activity was measured by an enzymatic rate method (24) without pyridoxal-5’-phosphate for AST activation. Alanine aminotransferase (ALT) activity was measured by an enzymatic rate method based on the International Federation of Clinical Chemistry (IFCC) method (25) without adding pyridoxal-5’-phosphate. The removal of NADH was measured spectrophotometrically at 340 nm. Glutamate dehydrogenase (GLDH) activity was measured by an enzymatic rate method, using a kit supplied by Roche Diagnostics.

### IL-12 *in vitro* bioactivity reporter assays

A HEK-Blue™ assay was used to prove IL-12 bioactivity *in vitro*. HEK-Blue™ IL-12 cells (InvivoGen, #hkb-il12) are designed to detect bioactive human and murine IL-12. To show *in vitro* bioactivity of IL-12 derived from AAV plasmids or AAV vectors, cells were cultured, transfected with plasmids or transduced with AAV vectors, and a reporter assay carried out according to manufacturer’s instructions. To show bioactivity of IL-12 expressed from the AAVs *in vivo*, plasma obtained from AAV-injected mice was examined in the HEK-Blue™ assay.

### Quantification of AAV vector genomes in tissue

Tissue samples were snap-frozen immediately after dissection. For DNA isolation, samples were homogenized in 900 µL RLT buffer (79216, Qiagen) + ß-mercapto-EtOH (1%), using a Precellys-24 homogenizer. Afterwards, samples were immediately placed on ice and subjected to Phenol-chloroform-isoamyl alcohol (77617, Sigma Aldrich) extraction. DNA was purified using the KingFisher (ThermoFisher) with the MagMAX DNA Multi-Sample Ultra 2.0 Kit (A45721). AAV vector genomes were detected after DNA extraction via singleplex ddPCR using the Automated Droplet Generator, QX200 Droplet Digital PCR System, and QX200 Droplet Reader (all Bio-rad). A custom TaqMan gene expression assay (#APDJ4HP; Thermo Scientific) was used as to detect the target region in the LP1 promoter. Oligonucleotide primers 5′-GGGAATGACTCCTTTCGGTAAGTG-3′ (forward) and 5′- CGCCCGCCTACGCT-3′ (reverse) and 5′-CCTGGGCAGTGTACAGCT-3′ (probe) were utilized for this assay.

### Histology and immunohistochemistry

Tissue samples of mouse livers were fixed in 4% PFA and paraffin embedded (i.e., formalin-fixed and paraffin embedded, FFPE). 3 µm thick sections of FFPE tissue on HistoBond+ slides (Marienfeld GmbH, Germany) slides were deparaffinized and rehydrated by serial passage through changes of xylene and graded ethanol for subsequent hematoxylin-eosin (H&E) and immunohistochemistry (IHC) staining. H&E staining was performed according to standard protocols (26). For the tolerability and efficacy study IHC was carried out on the automated Leica Bond™ platform (Leica Biosystems, Melbourne, Australia). Antigen retrieval was performed by incubating the sections in Leica Bond Epitope Retrieval Solution 1 (Citrate based, pH6, Cat# AR9961) for 30 minutes at 95°C for staining of CD45 and CD3 positive cells. For detection of CD8a positive cells antigen retrieval with Leica Bond Epitope Retrieval Solution 2 (EDTA based, pH9, Cat# AR9640) for 20 minutes at 95°C was performed. Sections were incubated with an anti-CD45 antibody (abcam, ab10558, rabbit polyclonal), anti- CD3 (abcam, ab5690, rabbit polyclonal), or an anti-CD8a antibody (abcam, ab209775, rabbit monoclonal, clone EPR20305), respectively. The antibodies were diluted (1:400, 1:200, or 1:1000, respectively) with Leica Primary Antibody Diluent (AR9352; Leica Biosystems, Nussloch, Germany) and incubated for 30 min at room temperature. In the experiment assessing the immune response induced by AAV.RS-mIL-12-noCpG, anti-CD8 detection was done on the Leica Bond RX using ER2 antigen retrieval, using D4W2Z clone at 1:800 dilution. anti-pSTAT4 IHC was done on the Ventana Discovery Ultra, using CC1 as antigen retrieval, using D2E4 clone at 1:400 dilution. Bond Polymer Refine Detection Kit (Cat# DS9800) was used for detection (3,3´Diaminobenzidine as chromogen, DAB) and counterstaining (hematoxylin).

### Image analysis

For analysis, liver sections were scanned with an Axio Scan.Z1 whole slide scanner (Carl Zeiss Microscopy GmbH, Jena, Germany) using an 20x objective (0.22 µm/px) in bright field illumination. Tumor sizes were calculated on HE stained sections using the image processing software HALO^®^ (Indica Labs, Corrales, NM, USA). A classifier based on DenseNET (27) was trained with sample regions from background, healthy and cancer tissue. For quantitative analysis of infiltrating cells, the percentage of the cells positive for CD45, CD3, CD8a, or pSTAT4, was calculated using the image processing software HALO 3.1 with CytoNuclear v2.0.9 module. Cell count analysis was then used to determine immunoreactive cells in tumor areas and normal tissue segmented in a pre-processing step using the trained classifier.

### Transfection and transduction of cell lines

HEK293H and HepG2 cells were cultured in DMEM + GlutaMAX + 10% FCS at 37°C. 25,000 HEK293 cells per 96-well were seeded 24 h prior to transfection using the Lipofectamine-3000 kit (Invitrogen). Transduction of HepG2 cells was performed at MOI 100,000 and the media was not replaced before the end of the experiment (72 h). Tetracycline (Tet-HCl, Sigma-Aldrich) was added to the cells 1-2 h after transfection and simultaneously to AAV addition in case of transduction.

### Pharmocokinetics/pharmacodynamics modeling

The dynamics of AAV, tet and tumor growth were fitted using a compartmental model that was run in three sequential steps. The first step was fitting the pharmacokinetic (PK) parameters of tet. In the second step, the effect of tet upon the activation of IL-12 was modeled by fitting the data for multiple doses of AAV given in the presence and absence of tet including both the constitutively active and riboswitch-controlled types of the AAVs. Finally, the IL-12 tumor dynamics were fitted by fixing the tet and tet-specific parameters in the full model while estimating the growth rate, kill rate and EC50 of riboswitch-controlled IL-12 against the tumor as shown in the equations below. The final model was constituted of 10 differential equations as follows (Equations 1–10):


(1) $$\frac{d_{AAVDose}}{d}\;=\;AAVDose\;*\;0$$

(2) $$\frac{d_{gut}}{dt}=-k_{abs}\cdot gut$$

(3) $$\frac{d_{plasma}}{dt}=k_{abs}\cdot gut-plasma\;\cdot \left(\frac{CL/F+Q_{1}/F}{V_{c}/F}\right)+\;\frac{Q_{1}/F}{V_{p1}/F}\cdot periph$$

(4) $$\frac{d_{periph}}{dt}=plasma\;\cdot \;\frac{Q_{1}/F}{V_{c}/F}-\;\frac{Q_{1}/F}{V_{p1}/F}\cdot periph$$

(5) $$TET_{conc}=\frac{plasma}{V_{c}/F}$$

(6) $$Tet_{effect}=\;TET_{conc}^{h}\;\frac{TETconc^{h}}{TETconc^{h}+EC_{50}Tet^{h}}$$

(7) $$\frac{dIL12\_a}{dt}=\frac{AAVDose\;\cdot kin\;}{AAVDose+\;EC_{50}\left(off\right)}+\frac{AAVDose\;\cdot kin\;}{AAVDose+\;EC_{50}\left(on\right)}\cdot Tet_{effect}-\;k_{out}\cdot IL12\_a$$

(8) IL12 = IL12_A + background

(9) $$\frac{d_{effectcomp}}{dt}=\;k_{ein}\cdot IL12-\;k_{eout}\cdot effectcomp$$

(10) $$\frac{d_{cancer}}{dt}=\;k_{growth}\cdot cancer\;\cdot \;\frac{1-cancer}{10^{5}}-\;\frac{E_{max}\cdot effectcomp}{EC_{50}cancer+\;effectcomp}\cdot cancer$$

Where *AAVDose* is the dose of AAV administered in the mouse; *gut* is representing the mass of administered tet in the gut; *k_abs_* is the rate of absorption of tet to plasma per hour; *CL/F* is the estimated oral clearance of Tet from the systematic circulation; *V_c_/F* is the volume of distribution of tet; *V_p1_/F and Q_1_/F* are the peripheral volume of distribution and intercompartmental clearance of tet, respectively; *periph* represents the mass of tet in the peripheral compartment; *TET_conc_* is the concentration of tet in plasma; *k_in_* is the maximal rate of generation of IL-12 and *k_out_* represents the natural elimination rate of IL-12; *EC_50_tet* is the concentration of tet required to illicit 50% of the maximum stimulation of IL-12 production in the presence of the AAV; *EC_50_ (on)* and *EC_50_ (out)* are the concentrations of AAV required to achieve maximal stimulation of IL-12; *k_growth_* represents the growth rate of the tumor per hour; *k_kill_* is the maximal rate of tumor size reduction per hour; *EC_50_cancer* is half of the IL-12 concentration required to illicit the maximal kill rate; *k_ein_* is the transfer parameter of drug to the effect compartment; and *k_eout_* is the movement of drug back from the effect compartment to plasma. Data was fitted using RxODE/nlmixr within R(4.1.2). Data visualization was also performed via R using the ggplot2 package (3.4.0).

### Statistical analysis

Statistical evaluations were performed utilizing GraphPad Prism Version 9.5.0 (GraphPad Software, LCC). All data are expressed as mean ± SEM. A normal distribution for all variables and equal variances across groups was assumed, and an unpaired two-tailed t-test against the specified group for statistical comparisons was executed. No alpha correction was implemented. The significance level (alpha) was set at 0.05, thus p-values less than 0.05 were considered statistically significant and marked with *, ** and, *** for p < 0.05, < 0.01 and < 0.001 respectively.

## Results

### Tetracycline-dependent control of IL-12 expression *in vitro* and *in vivo*

**Figure 1:**
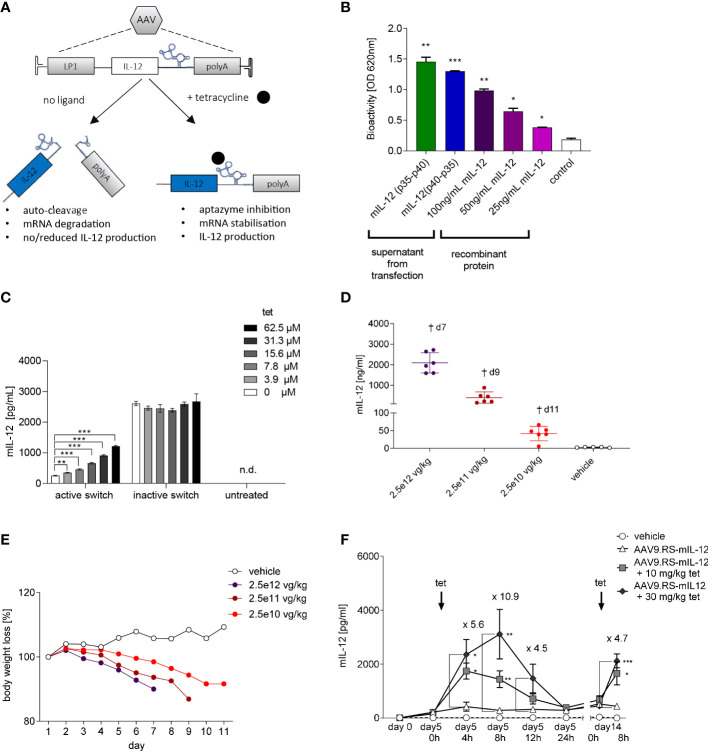
Riboswitch-controlled expression of single chain IL-12. **(A)** Schematic of AAV-mediated and tet-controlled expression of IL-12. **(B)** Bioactivity of single-chain murine IL-12 protein (mIL-12) produced by transfected HEK293 cells. Statistical analysis was conducted comparing groups to non-transfected HEK293 cells (control). **(C)** Tet concentration-dependent mIL-12 production in Hep G2 liver cells. Hep G2 cells were transduced with AAV9 harboring riboswitch-controlled mIL-12 cassette = active switch. Activation of riboswitch was assessed by comparing groups to no tet control (active switch, 0 µm). N= 3 biological replicates. **(D)** Application of mice with AAV9 for constitutive expression of murine IL-12 led to AAV dose-dependent increase of mIL-12 in plasma and **(E)** weight loss of mice. N= 6 animals per group. **(F)** Tet exposure-dependent mIL-12 production in C57BL/6NCrl mice. Mice were treated with either vehicle or 10^9^ vg/kg body weight of AAV9 delivering riboswitch-controlled mIL-12 sequence (AAV9.RS-mIL-12). On day 5 and day 14 riboswitch was activated with 10 or 30 mg/kg tet and mIL-12 levels were assessed at the indicated time points in plasma. Statistical analysis was performed comparing to AAV group receiving no tet (AAV9.RS-mIL12). N= 5 animals per group.

We have previously shown that the tet-dependent ribozyme K19 has the potential to control AAV-mediated reporter gene expression in mice using a hepatotropic AAV capsid and the hepatocyte-specific LP1 promoter (12). Features of this riboswitch-based expression control system include potent regulation, reversibility, and repeatable induction. Given the high liver exposure of tet, we hypothesized that this gene switch would enable liver-directed, inducible IL-12 expression following AAV-mediated gene delivery and allow for fine-tuned local immunotherapy of liver cancer. **Figure 1A** illustrates that vectorized IL-12 mRNA is stabilized following binding of tet by K19, resulting in onset of gene expression and subsequent therapeutic effects. In the absence of the ligand, basal expression is reduced due to K19 auto-cleavage and mRNA degradation. In a pilot experiment, we transfected HEK293 cells with plasmids expressing codon-optimized single-chain mIL-12 p40-linker-p35 or the p35-linker-p40 orientation and confirmed presence of bioactive IL-12 in the supernatant from either transfectant (**Figure 1B**). The p40-linker-p35 orientation has a superior stability (18) and was therefore used in all further experiments. We then cloned the mIL-12 gene into an AAV construct with the LP1 promoter and the K19 riboswitch in the 3’-UTR, and packaged the entire cassette into AAV9 (AAV9.RS-mIL-12). A control vector harboring mIL-12 and a catalytically inactive K19 switch (AAV9.mIL-12), was used to achieve constitutive IL-12 expression. We then transduced the human liver cancer cell line HepG2 with AAV9.RS-mIL-12 and observed that the highest tet-dose induced a 4.8-fold increase in mIL-12 production, reaching 45% of the expression levels mediated by the constitutively active AAV9.mIL-12 control (**Figure 1C**).

The encouraging *in vitro* biopotency data were the basis for a pilot *in vivo* dose-response study using AAV9.mIL-12. In this experiment, we delivered three different doses (2.5x10^10^, 2.5x10^11^, 2.5x10^12^ vg/kg) to achieve constitutive mIL-12 expression and plasma exposure in C57BL/6NCrl mice. All mice in the AAV9.mIL-12 groups lost body weight in a dose-dependent manner that correlated with IL-12 levels (**Figures 1D, E**). The low-dose group displayed 48 ng/mL IL-12 in plasma, which was not tolerated, suggested by body weight loss starting five days following vector administration. Body weight loss can be interpreted as a pathological consequence of circulating IL-12 levels that originate from hepatocytes, and show the need for a gene switch with low background expression. The low vector dose of 2.5x10^10^ vg/kg was selected for a PK study on tet-induced IL-12 expression in naïve mice (**Figure 1F**). The study design included three groups of mice receiving AAV9.RS-mIL-12 or saline, and tet at 10 mg/kg or 30 mg/kg, on day 5 and day 14 (tet re-challenge) following vector delivery. At 8 h after the tet re-challenge, tet concentrations in plasma were 100 nM and 750 nM in the 10 mg and 30 mg tet groups, respectively. The plasma IL-12 levels showed tet dose-dependent induction. 30 mg/kg tet induced IL-12 almost 11-fold after 8 h over background levels (day 5, 0 h). Following the fast on-kinetic, IL-12 levels had returned to baseline after 24 h. The tet re-challenge induced IL-12 at a lower level of 4.7-fold, and the absolute IL-12 concentrations ranged between 2-3 ng/mL. In summary, these data showed repeatable and quick ON and OFF kinetics for tet-induced IL-12 expression *in vivo* following systemic AAV9.RS-mIL-12 delivery.

### AAV9.RS-mIL-12 dose finding in healthy mice

**Figure 2:**
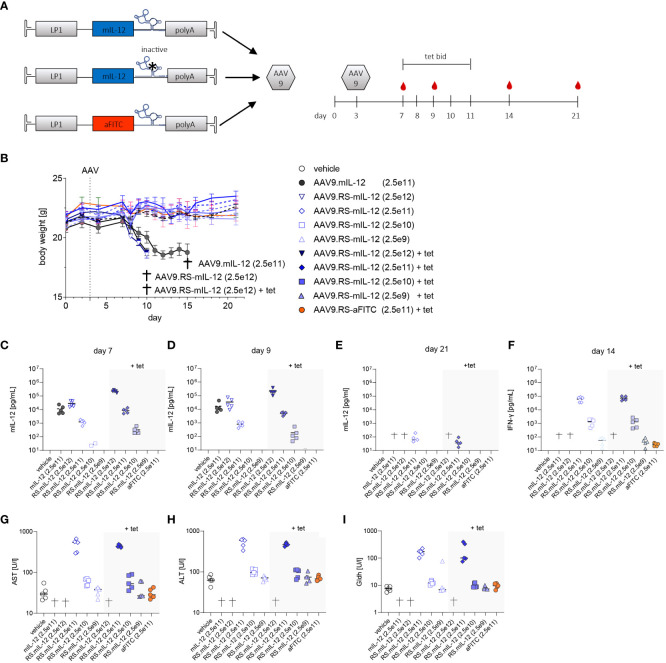
*In vivo* dose finding for AAV9-mediated and tetracycline-controlled mIL-12. **(A)** Schematic designs of applied cassettes; active (AAV9.RS-mIL-12), inactive switch (AAV9.mIL-12) and aFITC nanobody harboring vector control (AAV9.RS-aFITC) and experimental schedule for application of AAV, tet, blood sampling (red droplet). **(B)** Body weight development of AAV +/- tet treated C57BL/6NCrl mice. Groups that reached pre-defined humane endpoints are indicated by †. Weights are given as mean of groups and AAV doses are presented in vg/kg body weight. **(C-E)** Development of mIL-12 levels in plasma was assessed on days 7 **(C)**, 9 **(D)** and 21 **(E)**. **(F)** IFNγ levels in plasma were measured on day 14. **(G-I)** Level of liver enzymes – aspartate aminotransferase (AST), alanine aminotransferases (ALT) and glutamate dehydrogenase (GLDH) were assessed on final day of experiment. AAV doses are given as vg/kg body weight, N= 5 animals per group, tet bid = tet twice daily 30 mg/kg, Symbols shown only for values >10 pg/ml.

Next, we investigated RS-controlled IL-12 expression by multi-day tet exposure (**Figure 2A**). The aim of this study was to identify a vector dose that allowed for tet-dependent sustained induction of relevant IL-12 levels in plasma during tet administration, but full reversal to background levels upon tet retraction. Weight loss and liver enzymes were monitored as a measure of toxicity. The study design entailed delivery of AAV9.RS-mIL-12 at doses from 2.5x10^9^-2.5x10^12^ vg/kg. Half of the animals of each group were subjected to twice daily (b.i.d.) tet applications for 5 consecutive days, while the other half received no tet. The body weight development was normal for all groups except the highest AAV9.RS-mIL-12 dose groups (with or without tet) and the constitutive AAV9.mIL-12 group (**Figure 2B**). This observation suggests that tolerated IL-12 levels were achieved in most dose groups. IL-12 levels in plasma showed vector dose-dependency during and after tet-induction (**Figures 2C, D**). Importantly, in the 2.5x10^10^ vg/kg dose group that had received tet, IL-12 levels had returned to background three days after the final tet exposure (**Figure 2E**). The same treatment group showed elevated levels of IFNγ, the key effector of IL-12-induced T-cell activation (**Figure 2F**). Liver enzymes of animals that received the 2.5x10^10^ vg/kg dose were not elevated at any time point of our investigation, independent of tet treatment (**Figures 2G–I**). In summary, the dose of 2.5x10^10^ vg/kg had the best combination of PK and safety.

### Riboswitch-controlled IL-12 is safe and efficacious in a murine liver cancer model

**Figure 3:**
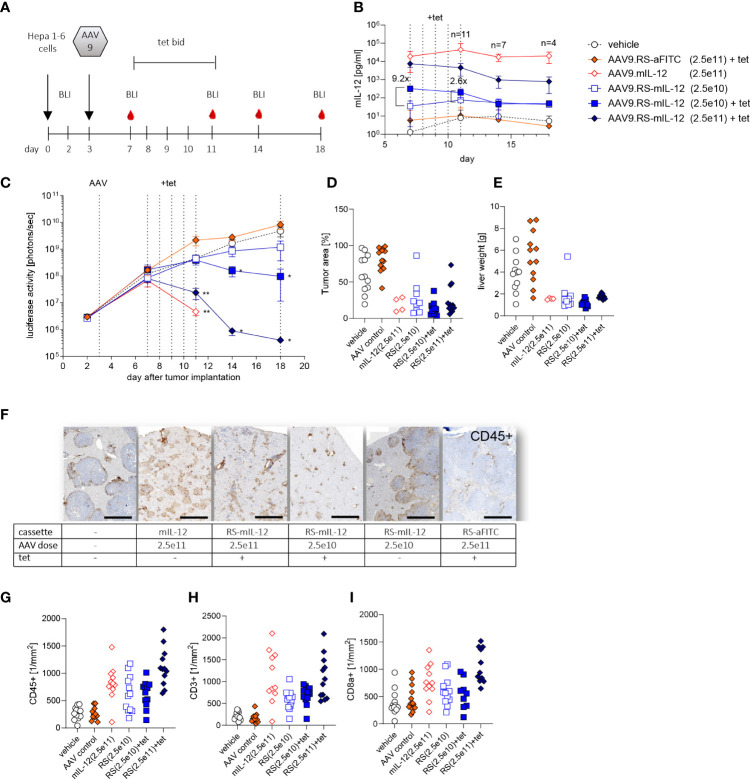
Tolerability and efficacy of riboswitch-controlled mIL-12 expression in murine liver cancer model. **(A)** Schedule of treatment and sample collection in mouse tumor study. On day 0 all animals received luciferase expressing Hepa 1-6 tumor cells, on day 3 AAVs were applied. BLI = time point of bioluminescence measurement for assessment of Hepa 1-6 tumor cell growth, tet bid = tet twice daily with 30 mg/kg, N=12 per group at start of experiment. **(B)** mIL-12 levels were assessed in plasma; indicated is the fold increase of mIL-12 after tet activation comparing the two groups receiving 2.5x10^10^ vg/kg of AAV9.RS-mIL-12 with or without activation by tet. AAV doses given as vg/kg body weight. **(C)** Tumor growth was analyzed via BLI over 18 days and treatment effects were assessed by comparison to vehicle group receiving no AAV. **(D)** Tumor growth was further assessed by histological analysis of liver tissue sections on final day of experiment. **(E)** As additional indicator for tumor growth total weights of livers were assessed. **(F)** Cellular immune response to treatment was assessed. Representative liver tissue sections stained for CD45+ cells are shown. Scale bar = 1 mm **(G-I)** Quantification of immune cells was conducted for CD45+ **(G)**, CD3+ **(H)**, and CD8+ **(I)** cells in liver sections.

To test whether HCC can effectively be treated with AAV9.RS-mIL-12, we used an orthotopic HCC mouse model. For this model, we generated a Hepa1-6 tumor cell line that stably expressed luciferase, so that tumor growth could be monitored by BLI *in vivo* over time. These tumor cells were injected on day 0 to form tumor nodules in the liver (**Figure 3A**). On day 3, virus preparations were injected intravenously. To induce transgene expression, tet was administered twice daily from day 7 to 11. Animals were kept for another week without tet and were euthanized for analysis on day 18.

To control for transgene induction, blood samples were taken and IL-12 levels in plasma were determined. Constitutive expression with 2.5x10^11^ vg/kg of AAV9.mIL-12 resulted in IL-12 plasma levels above 10 ng/ml at all time-points (**Figure 3B**). Like in the previous study in healthy mice, these levels led to body weight loss, starting around day 11 (**Supplementary Figure 1**), and 7 out of 12 mice were euthanized prematurely in this group over the course of the study. In animals treated with 2.5x10^11^ vg/kg of AAV9.RS-mIL-12, tet-induced IL-12 expression reached 7.5 ng/ml in plasma on day 7 which dropped below 1 ng/ml on days 14 and 18 after stopping tet administration. In this group, no body weight loss was observed except for one animal, indicating the increased safety profile of riboswitch-controlled IL-12 expression. In animals treated with a ten-fold lower vector dose (2.5x10^10^ vg/kg of AAV9.RS-mIL-12), basal expression of around 50 pg/ml IL-12 was observed in the absence of tet, which remained constant over time. Tet administration raised IL-12 levels to 318 pg/ml, which dropped to ~50 pg/ml after ceasing tet treatment. In the control groups (vehicle or AAV9.RS-aFITC), IL-12 levels remained below 10 pg/ml. These data confirmed that the IL-12 level was dependent on the vector dose and could be induced by tet in animals treated with AAV9.RS-mIL-12.

Tumor growth was monitored by BLI (**Figure 3C**). In control groups, tumor burden increased continuously. In animals with constitutive IL-12 expression, after initial tumor growth, tumor size declined until the animals had to be euthanized due to body weight loss. Similarly, induction by tet on day 7 in animals with riboswitch-controlled IL-12 led to an AAV-dose-dependent reduction of tumor burden with almost complete regression in the high dose group. In the absence of tet, only a moderate effect on tumor growth inhibition was observed.

At the end of the experiment, tumor burden was also determined by quantifying the tumor area in histological liver sections (**Figure 3D**) and by measuring liver organ weight, comprised of liver and tumor tissue (**Figure 3E**). These data were in line with the BLI measurements.

Immunohistological analysis of liver sections revealed that IL-12 expression resulted in an increase of immune cells (**Figures 3F–I**). The number of cells depended on the IL-12 expression level. A large fraction of the infiltrated cells were CD3^+^ and CD8^+^ T cells.

Taken together, these results suggest that IL-12 expression led to recruitment of cytotoxic T cells, which controlled tumor growth, and that riboswitch-regulated IL-12 was safe and efficacious.

### Optimization of human IL-12 AAV vector

**Figure 4:**
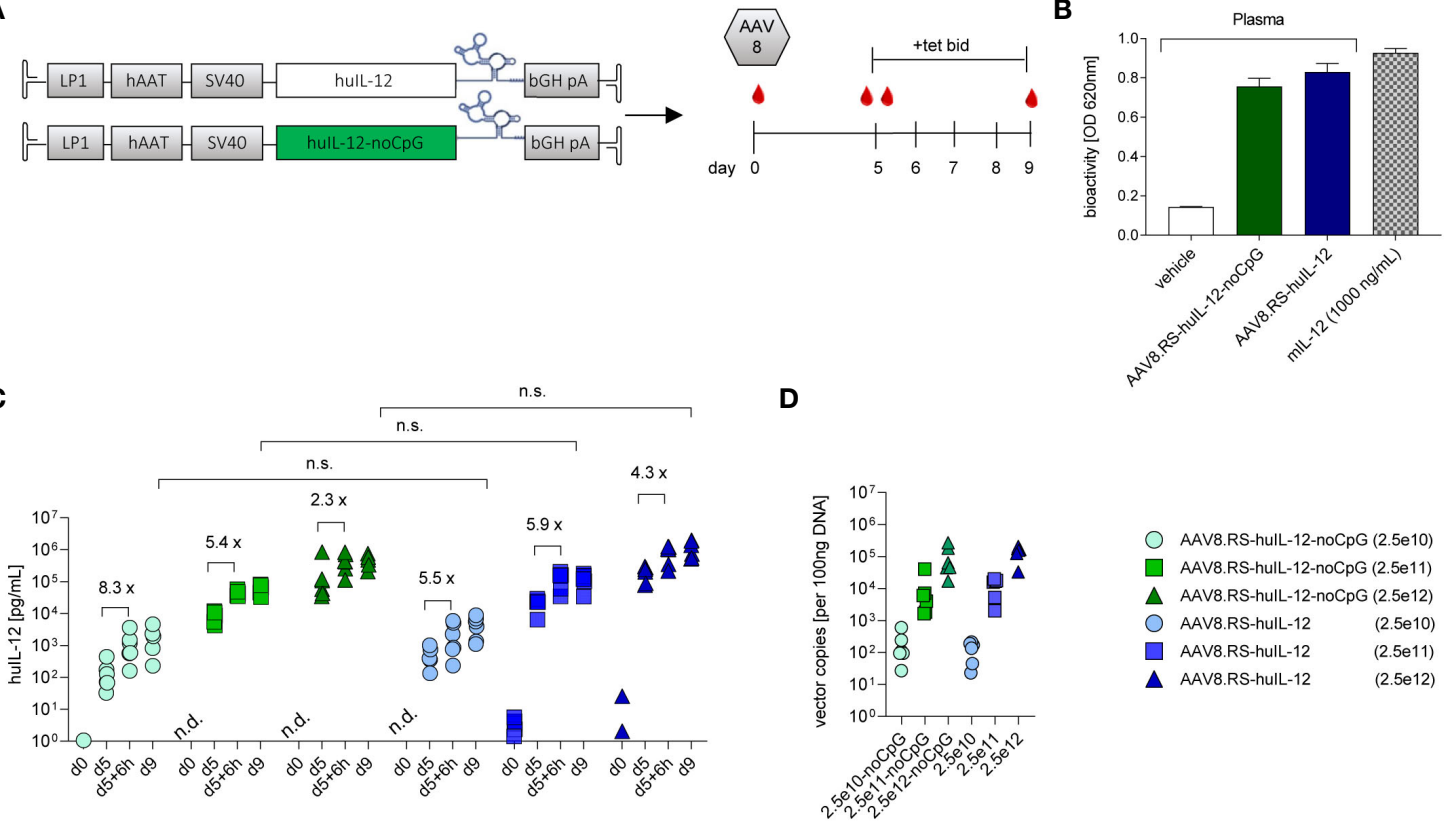
Optimization of human IL-12 (huIL-12) AAV vector candidate. **(A)** Schematic of CpG containing (huIL-IL12) and CpG-depleted (huIL-12-noCpG) riboswitch-controlled expression cassettes for human IL-12 (huIL-12), and respective study design for their comparison in C57BL/6NCrl mice. **(B)** Bioactivity measurement of IL-12 in plasma of mice injected with AAV8.RS-huIL-12-noCpG or AAV8.RS-huIL-12. **(C)** Expression efficacy of optimized sequences were compared by measurement of huIL-12 protein levels in plasma on day 9, 6h after tet activation. n.s., non-significant. Riboswitch activation effects were assessed comparing huIL-12 levels before and 6h after tet treatment on day 5. Symbols shown only for values >1 pg/ml. n.d. not detectable. **(D)** Transduction efficacy was assessed by quantification of vector genomes in liver tissue. AAV doses = vg/kg body weight, tet bid = tet twice daily 30 mg/kg, N=6 per group,.

We then performed AAV cassette optimization by combining genetic elements that have been reported to result in beneficial AAV-mediated transgene expression in hepatocytes of both animals and human subjects (19, 28, 29). The introduction of an intron and a 5’-UTR element to the AAV cassette increases transgene product levels *in vivo* (29). Therefore, we adopted a clinically validated cassette design including the LP1 promoter, an SV40 intron (19), the coding sequence of huIL-12 (p40-linker-p35), the K19 Tet-switch, and the bovine growth hormone pA sequence (pAAV.RS-huIL-12) (**Figure 4A**). The cellular Toll-like receptor 9 (TLR9) recognizes unmethylated cytosine-phosphate-guanosine motifs (CpG) motifs in the therapeutic expression cassettes packaged in an AAV capsid and induces innate and adaptive immunity, thereby limiting the duration of gene therapy (20). We therefore created an equivalent AAV-construct to pAAV.RS-huIL-12 in which CpGs of the IL-12 coding sequence were depleted (pAAV.RS-huIL-12-noCpG). AAV8 shows less extrahepatic biodistribution than AAV9 (30). Therefore, it is more suitable for liver-restricted gene delivery, which is required for our concept of vectorized IL-12 immunotherapy. To characterize both designs, AAV.RS-huIL-12 and AAV.RS-huIL-12-noCpG were packaged as AAV8 vectors and delivered to C57BL/6NCrl mice at a single dose that resulted in tet-dependent induction of bioactive huIL-12 (**Figure 4B**). We then performed a dose escalation study using both AAV8.RS-huIL-12 and AAV8.RS-huIL-12-noCpG in C57BL/6NCrl mice and observed no difference in the performance of the two designs as assessed by the huIL-12 levels in plasma (**Figure 4C**). Vector genome analysis in liver confirmed comparable transduction of dose-matched groups (**Figure 4D**). Based on these data, we nominated AAV8.RS-huIL-12-noCpG as the lead candidate and used the mouse surrogate (AAV8.RS-mIL-12-noCpG) for subsequent pre-clinical PK/PD experiments as huIL-12 does not act on murine cells (31).

### AAV8.RS-mIL-12-noCpG is efficacious, and IL-12 levels correlate with efficacy

**Figure 5:**
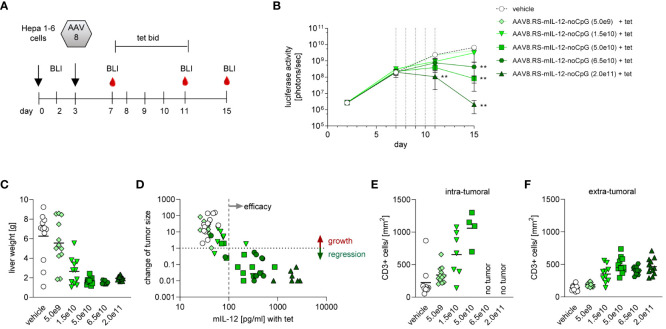
Dose finding with AAV8.RS-mIL-12-noCpG in HCC model. **(A)** Treatment and sampling schedule for analysis of AAV8.RS-mIL-12-noCpG in C57BL/6NCrl mice. Animals were treated with luciferase expressing Hepa 1-6 tumor cell on day 0, AAV was administered on day 3 and riboswitch was activated with 30 mg/kg body weight of tet from day 7 to 11, twice daily. **(B)** Tumor growth was assessed via luciferase activity measurement on day 3, 7, 11 and 15. Statistical effects were assessed comparing signals of treatment group to signals of vehicle treated group. N=12 per group. **(C)** As a second indicator for tumor growth liver weight was measured. **(D)** Effects of mIL-12 levels on tumor growth were visualized by plotting mIL-12 plasma levels vs changes of tumor size for individual animals. Change of tumor size was calculated by comparing luciferase signal on day 15 (end of experiment) vs day 7 (start of tet treatment). **(E, F)** Quantitative analysis of CD3 staining of liver tissue sections; CD3+ cells in tumor tissue **(E)**, and in non-tumor liver tissue **(F)**.

To confirm the efficacy of the mouse surrogate of the lead candidate and to determine efficacious doses, we titrated AAV8.RS-mIL-12-noCpG in the orthotopic HCC mouse model (**Figure 5A**). All animals received tet from day 7 to 11 after tumor implantation. The level of induced IL-12 in plasma was dependent on the AAV dose and dropped to pre-tet levels after ceasing tet administration (**Supplementary Figure 2**). Tumor growth inhibition depended on the AAV dose with complete responses in all animals of the highest dose group (**Figure 5B**). Doses above 5.0x10^10^ vg/kg resulted in >90% of tumor growth inhibition with all animals responding. Treatment with 1.5x10^10^ vg/kg resulted in tumor growth inhibition of ~50%. In the lowest dose group (5.0x10^9^ vg/kg), only minimal IL-12 expression was detected with almost no tumor growth inhibition. Tumor burden was also determined by measuring liver organ weight (**Figure 5C**) which confirmed the BLI measurements. Because of the variability between animals of the same dose group, we analyzed the relationship of IL-12 levels and tumor growth in individual animals (**Figure 5D**). Interestingly, there was a clear correlation between the level of IL-12 induced by tet and the reduction of tumor BLI signal. In general, induction of IL-12 to more than 100 pg/ml in plasma resulted in tumor regression, whereas tumors continued to grow in animals with lower levels. To investigate the T cell response, immunohistological analysis of liver sections was performed at the end of the study. The number of CD3^+^ T cells was quantified separately within or outside of the tumor area (**Figures 5E, F**). In the vehicle-treated group, a low number of T cells was seen inside and outside of the tumor. Induction of IL-12 by tet from days 7-11 in the riboswitch-controlled group resulted in dose-dependent increase of intratumoral CD3^+^ cells on day 15, suggesting the activation of an anti-tumor T cell response. Importantly, outside of the tumor tissue (in liver parenchyma), CD3^+^ cell numbers remained low in all groups. These results confirmed that the mouse surrogate of the lead candidate (AAV8.RS-mIL-12-CpG) showed efficacy at doses ≥1.5x10^10^ vg/kg and that IL-12 levels correlated with tumor growth inhibition.

### IL-12 induces T cell infiltration preferentially into the tumor and persistently activates the immune response

**Figure 6:**
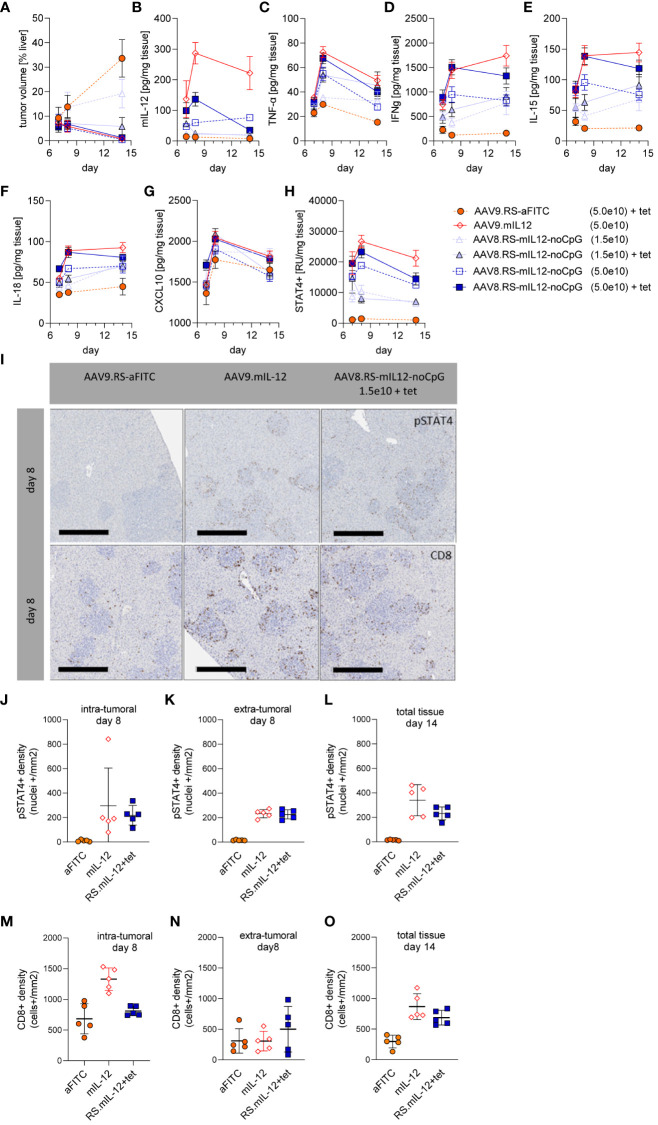
Immune response induced by AAV8.RS-mIL-12-noCpG in HCC model. In the HCC model, mice were treated with tumor cells on day 0, AAVs on day 3, and 30 mg/kg tet twice daily on days 7-11. Groups of mice were euthanized for analyses on day 7, 8 or 14 after tumor inoculation and **(A)** tumor volume, and levels of **(B)** mIL-12, **(C)** TNF-α, **(D)** IFNγ, **(E)** IL-15, **(F)** IL-18, **(G)** CXCL10 and **(H)** pSTAT4 were assessed in liver tissue. **(I)** Representative pictures of immunohistologically stained liver sections for pSTAT4+ cells (upper row) or CD8+ cells (lower row), 8 days post tumor inoculation, scale bar = 400 µm. Quantitative analysis of histological stained liver tissue for pSTAT4+ nuclei on day 8 **(J)** intra-, or **(K)** extra-tumoral, and **(L)** of total tissue on day 14. Analysis of liver tissue stained for CD8+ cells on day 8 **(M)** intra-, or **(N)** extra-tumoral or **(O)** of total tissue on day 14. N=5 per group and time point, AAV doses = vg/kg body weight.

To analyze the immune response in more detail, we performed a dedicated study with AAV8.RS-mIL-12-noCpG in the orthotopic HCC model with the same schedule as before and euthanized animals for specimen collection at three different time-points: on day 7 (after first tet administration), day 8, and day 14 (three days after the last tet dose). The tumor area was quantified in histopathological liver sections (**Figure 6A**). As in the previous experiment, IL-12 – either expressed constitutively or induced by tet – resulted in reduction of tumor size. In the lower AAV dose group (1.5x10^9^ vg/kg), induction was necessary for efficacy, whereas in the higher dose group (5.0x10^9^ vg/kg) basal IL-12 expression already induced tumor reduction. Quantification of IL-12 levels in plasma confirmed highest IL-12 levels by constitutive expression, AAV dose dependency and inducibility of IL-12 levels by tet (**Supplementary Figure 3**). Cytokines were also quantified in liver lysates (**Figures 6B–G**). Without tet induction, IL-12 tissue levels remained constant over time and depended on the AAV dose as in plasma. Both the constitutive expression and the high dose group with tet displayed elevated IL-12 tissue levels on day 7, which further increased on day 8. Ceasing tet administration led to reduction of IL-12 tissue levels in the riboswitch-controlled group on day 14 as expected. The low dose AAV8-RS-mIL-12-noCpG group confirmed these patterns on days 7 and 14. However, the value on day 8 was unexpectedly low, which might be explained be a technical problem in the virus injection in these animals or the preparation of samples for analysis.

We confirmed that IL-12 expression induced IL-12 receptor signaling by determining the amount of phosphorylated STAT4 (pSTAT4) which was in line with IL-12 levels (**Figure 6H**). Interestingly, IFNγ, one of the downstream mediators of IL-12, was upregulated and remained high even after ceasing tet administration, suggesting a sustained immune response (**Figure 6D**). Other proinflammatory cytokines or chemokines followed similar patterns as IL-12 or IFNγ.

To investigate the location of IL-12R signaling, immunohistological analysis of liver sections was performed (**Figures 6I–L**). Staining for pSTAT4 confirmed that IL-12 induced persistent IL-12 receptor signaling which was absent in the control group at all time points. On day 8, pSTAT4 was detected to a similar degree within or outside of the tumor tissue area. On day 15, the tumor area was too small to quantify the intra- and extratumoral staining separately. To investigate the cytotoxic T cell response, CD8^+^ T cells were stained and quantified in the liver sections (**Figure 6I, M–O**). With constitutive IL-12 expression (AAV9.mIL-12) the number of intratumoral CD8^+^ T cells was increased compared to vector control on day 8. Induction of IL-12 by tet from day 7 in the riboswitch-controlled group did not yet elevate intratumoral CD8^+^ T cell numbers on day 8 but seemed to require a longer treatment. Although on day 15 tumor area was too small to quantify intra- and extratumoral staining, qualitatively there was a high CD8^+^ cell density at the regressing tumor lesions while the CD8^+^ cells in the liver parenchyma remained low.

### Tolerability of AAV8.RS-mIL-12-noCpG in healthy mice

**Figure 7:**
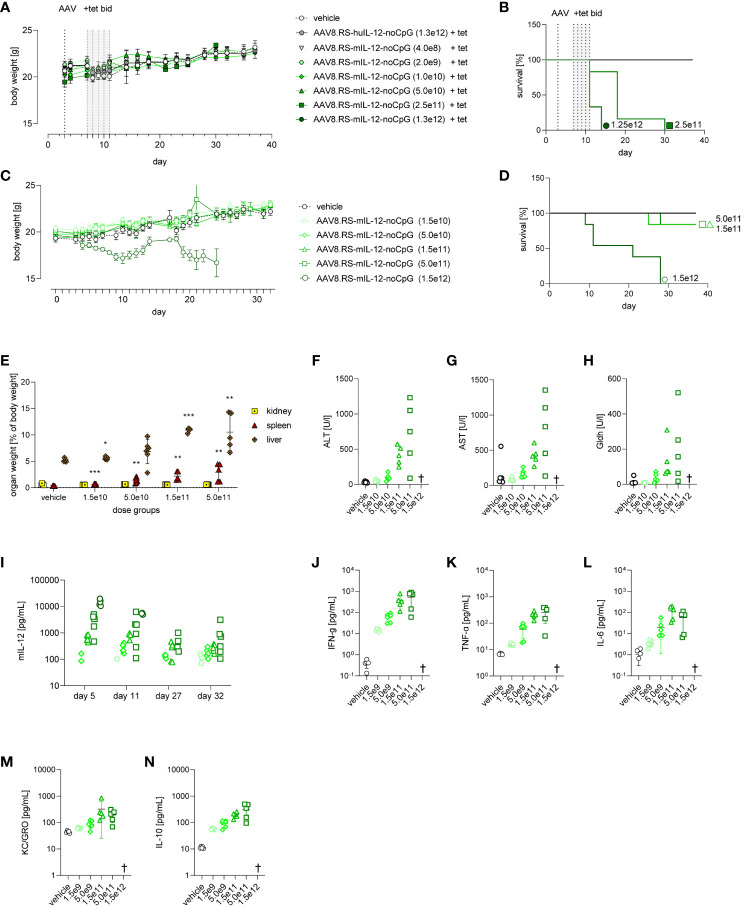
Tolerability of AAV8.RS-mIL-12-noCpG in healthy mice with and without tetracycline induction of mIL-12. **(A)** Body weight development of mice receiving 1.5x10^10^ to 1.5x10^12^ vg/kg of AAV8.RS-mIL-12-noCpG on day 3 and 30 mg/kg of tet twice daily on days 7-11. **(B)** Survival curves of AAV dose groups with tet-induced switch activation. **(C)** Body weight development of mice after AAV application on day 0 receiving no tet. **(D)** Survival curves of AAV dose groups receiving no tet. **(E)** Effects of AAV doses without tet on organ weights of kidney, spleen and liver weights, were assessed and statistically compared with respective organ weight of vehicle group. **(F)** Levels of aspartate aminotransferase (AST), **(G)** alanine aminotransferases (ALT), and **(H)** glutamate dehydrogenase (GLDH) were assessed on day 32 at the end of study. **(I)** mIL-12 plasma levels on day 5, 11, 27 and 32. Symbols shown only for values >10 pg/ml. On final day of study cytokine levels of **(J)** IFN*γ* **(K)** TNF-α and **(L)** IL-6, **(M)** KC/GRO, **(N)** IL-10, were measured in plasma. AAV doses = vg/kg body weight, tet bid = tet twice daily 30 mg/kg body weight, N=6 per group. † = euthanized groups. *p< 0.05, **p< 0.01; ***p< 0.001.

As toxicity is a major concern of IL-12 therapy, we analyzed in detail the tolerability of our vectorized and riboswitch-controlled IL-12 by administering a wide range of AAV8.RS-mIL-12-noCpG doses to non-tumor bearing, healthy mice. First, we studied an acute setting in which IL-12 was induced by tet using the same regimen as in the efficacy studies (**Figures 7A, B**). In the highest dose group (1.3x10^12^ vg/kg), all animals had to be euthanized between day 11 (last day of tet administration) and day 15 due to ataxia and/or poor general condition. These animals exhibited IL-12 levels in plasma above 45 ng/ml after induction (**Supplementary Figure 4**). Animals in the second highest dose group (2.5x10^11^ vg/kg) had to be euthanized about one week later mainly because of a distended abdomen due to ascites. Tet-induced IL-12 plasma levels were at 6.2 ng/ml, and liver enzymes were increased in most of the animals at the time of euthanasia. All doses ≤5x10^10^ vg/kg were well tolerated without adverse events. Also, in the long-term follow-up of these animals after stopping tet application, no adverse findings were observed.

As AAV8.RS-mIL-12-noCpG treatment resulted in expression of IL-12 even in the absence of tet, we performed a tolerability study in a chronic setting without induction by tet to determine which basal IL-12 levels were tolerated long-term (**Figures 7C–N**). Healthy mice were injected with a wide range of AAV8.RS-mIL-12-noCpG doses and monitored for 5 weeks. At the highest AAV dose used (1.5x10^12^ vg/kg), all animals lost body weight over the course of the study (**Figure 7C**) and had to be euthanized prematurely (**Figure 7D**). At 5.0 x10^11^ vg/kg and 1.5x10^11^ vg/kg, only one animal per group had to be euthanized after more than 3 weeks. All other animals did not display any macroscopic observations.

At the end of the study, several organs were preserved for further analysis. The organ weights of liver, spleen and kidney were determined (**Figure 7E**). Organ weights were not available for mice that underwent unscheduled early termination. Higher increases in spleen and liver weights were present at ≥1.5x10^11^ vg/kg in all animals followed by animals at 5.0x10^10^ vg/kg, and no changes in weights at 1.5x10^10^ vg/kg. Kidney weights were not affected at any dose level. A detailed histopathological examination revealed adverse microscopic findings in spleen, liver, and bone marrow in affected animals. In the liver, microscopic findings included minimal to moderate mixed cell inflammation, minimal to moderate hepatocyte degeneration, minimal to slight single cell necrosis, and minimal thrombosis. The spleen displayed minimal to severe decrease in lymphocyte cellularity, minimal to severe increase in cellularity of stromal cells, minimal thrombosis and minimal to severe extramedullary hematopoiesis. In the bone marrow, adverse microscopic findings were minimal to moderate decrease in erythroid and/or myeloid cellularity and/or minimal to severe necrosis. These findings were more prevalent and more severe in higher dose levels. In the group injected with 1.5x10^10^ vg/kg, findings were only of minimal severity grade with low incidences and were considered non-adverse. Liver histopathology changes correlated with an increase in plasma liver enzymes in a dose-dependent manner at the end of the study (**Figures 7F–H**).

We determined basal expression of IL-12 in the plasma over the course of the study (**Figure 7I**). Overall, the levels were dependent on the AAV dose as before and showed a small decline from day 5 to day 32. At doses ≤5.0 x10^10^ vg/kg, no or only minimal IL-12 plasma levels were detected. At the highest dose (1.5x10^12^ vg/kg), IL-12 levels on day 5 were >10 ng/ml (mean 14 ng/ml) and remained high until the day of early unscheduled termination. In the other groups, surviving animals had IL-12 plasma levels<5 ng/ml. At the end of the study, additional cytokines were increased in plasma dependent on the AAV dose and may indicate immune activation and a potential safety liability (**Figures 7J-N**).

In summary, we observed that high levels of IL-12 could induce several adverse findings, such as body weight loss, ascites, elevated liver enzymes and plasma cytokines, increased weight of liver and spleen, and histopathological changes in spleen, liver and bone marrow. Safety of AAV8.RS-mIL-12 was dependent on the dose with no findings at ≤1.5x10^10^ vg/kg and minimal findings at 5.0x10^10^ vg/kg.

### Increase of tet dose and PK/PD modeling

**Figure 8:**
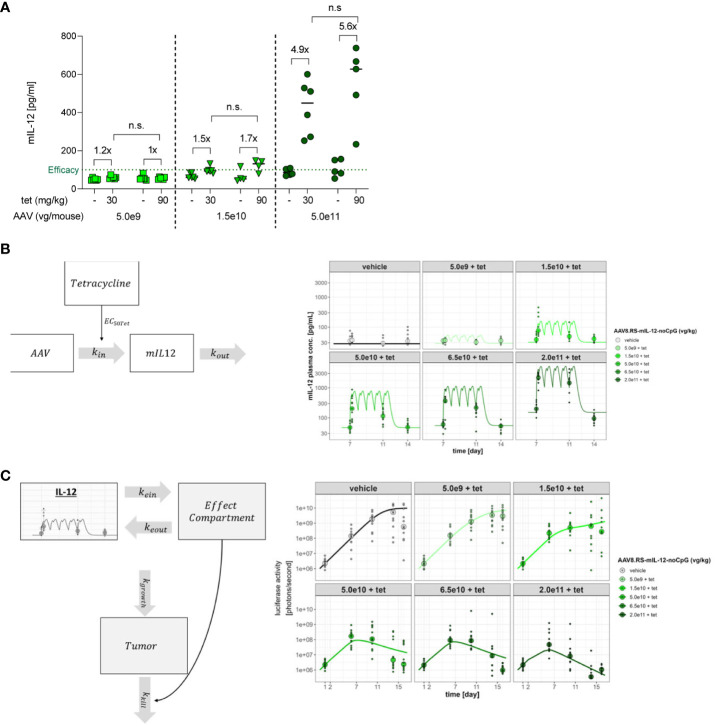
Induction of mIL-12 with different tetracycline doses and PK/PD modeling for IL-12 and tumor growth. **(A)** mIL-12 induction after tet doses of 30 or 90 mg/kg in C57BL/6NCrl mice receiving 5.0x10^9^, 1.5x10^10^, or 5.0x10^11^ vg/kg of AAV8.RS-mIL-12-noCpG. Plasma was collected 21 days post AAV application, before and 6 h after tet administration. N=6 per group. n.s., not significant **(B)** PK/PD model. The model is implemented in two stages. The first stage describes the relationship between the AAV dose, tet plasma exposure and levels of generated IL-12 over time according to the dosing intervals of tet. **(C)** The second stage of the model defines the relationship between generated IL-12 levels and tumor elimination. The model assumes an effect compartment to allow for the apparent delayed and prolonged effect of IL-12 upon the tumor where the drug moves to the effect compartment, guided by parameters k_ein_ and k_eout_. The effect of IL-12 on tumor size was assumed to follow an E_max_ relationship with a hill slope of 1. Growth of the tumor is defined by the capacity limited parameter k_growth_ while the tumor reduction is expressed by the parameter k_kill_ which reflects the maximal rate of tumor elimination. Plots in **(B, C)** are based on the treatment scheme used in tumor studies.

In our tumor and tolerability studies, tet has been used at a fixed concentration of 30 mg/kg. In **Figure 1D** we have observed that an increase of the tet dose from 10 mg/kg to 30 mg/kg led to higher IL-12 induction. Therefore, we tested whether an increase of the tet dose to 90 mg/kg would further improve the dynamic range of expression and hence, the induction window (**Figure 8A**). A clear induction of IL-12 by tet was only observed at the highest vector dose used (5.0 x 10^11^ vg/kg). Induction from baseline was 4.9-fold for 30 mg/kg tet and 5.6-fold for 90 mg/kg. This moderate difference indicated that at 30 mg/kg tet almost complete aptazyme inhibition and mRNA stabilization was achieved and that higher tet doses would not increase the therapeutic window.

PK/PD modeling was performed to quantify the potency of AAV.RS-mIL-12 + tet in mice and its effect on tumor growth (**Figures 8B, C**). The modeling allowed for parameterizing the effect of AAV to optimize dosage and to estimate with precision the minimum required IL-12 level to achieve activity as well as the level of tet required to fully activate IL-12 expression (**Table 1**). The required tet exposure was calculated to estimate whether the antibiotic can be given at safe doses in humans given its known clinical PK profile. Modelling showed an EC50 of 348 nM for tet in mice and, thus, demonstrated that safe doses of the drug can be given clinically to activate AAV.RS-IL-12. The modeling also revealed a ~15-fold higher potency of AAV.RS-IL-12 in presence of tet compared to absence of tet, confirming that induction of IL-12 by tet increases efficacy. With this complex PK/PD relationship being established, it is possible to translate the *in vivo* activity in mice to predicted human activity. However, a few caveats remain in the translation which include the uncertainty about the innate turnover levels of IL-12 in mice and humans and any variables that might change the response of a tumor to murine or human IL-12.

**Table 1:** Estimated parameters from the models used to fit the PK of tet, the dynamics of tetracycline activation of AAV and the AAV stimulation of IL-12 production and the pharmacodynamic model describing the relationship between IL-12 production in plasma and its effect on tumor size reduction.

Parameter	Value [% RSE]
Tet PK	
***k_abs_ [h^-1^]***	0.83 [108%]
***CL/F [L/h/kg]***	3.33 [13.6%]
***V_c_/F [L/kg]***	1.59 [119%]
***V_p1_/F [L/kg]***	2.37 [41%]
***Q_1_/F [L/h/kg]***	0.605 [46.5%]
Tet/IL-12 dynamics	
***k_out_ [h^-1^]***	0.208 [20.6%]
***k_in_[h^-1^]***	k_out_ x 10^5^
***EC50_tet_ [nM]***	348 [19%]
***EC_50_ (on) [AAV units]***	25.4 x 10^9^ [4.6%]
***EC_50_ (off) [AAV units]***	375 x 10^9^ [6.6%]
IL-12 - Tumor Dynamics	
***k_growth_ [h^-1^]***	0.0454 [0.39%]
***k_kill_ [h^-1^]***	0.0658 [2.7%]
***EC_50_cancer [pg/mL]***	0.560 [0.45%]
***k_ein_ [h^-1^]***	0.0728 [2.33%]
***k_eout_ [h^-1^]***	k_ein_/20

## Discussion

HCC and CRC with liver metastasis are indications with high unmet medical need. For a long time, the highly proinflammatory cytokine IL-12 has been proposed for anti-tumor therapy, but its therapeutic potential has not been realized due to severe systemic toxicity. Therefore, we aimed to explore whether liver tumors can be treated safely by an AAV-based gene therapy that enables tissue-specific and regulatable expression of IL-12.

Using a combination of hepatotropic AAV8 vectors, the liver-specific promotor LP1 and the K19 riboswitch, we achieved tet-dependent control of IL-12 expression *in vitro* and *in vivo*. In an orthotopic liver tumor model, AAV-mediated expression of IL-12 induced IL-12R signaling (STAT4 phosphorylation), and led to IFN-γ production, infiltration of T cells and to tumor regression in a vector dose-dependent manner. IL-12 activated the immune response persistently even after removal of tet and induced T cell infiltration or accumulation preferentially into the tumor, but not into liver parenchyma. Therefore, we hypothesize that IL-12 initiates the immune response, but additional factors are required for a full and sustained anti-tumor response, such as presence of tumor antigens which can stimulate T cells and lead to their proliferation. One limitation of our studies was that the potential of abscopal responses or long-term immunity was not tested in a tumor re-challenge model. However, long-lasting systemic efficacy appears likely, given positive data from several clinical and non-clinical IL-12 gene therapy studies (7, 11, 32). One disadvantage of tet is its antibiotic activity, which potentially comes with side effects. However, our treatment concept embodies a limited tet delivery of days to weeks at a time that will be sufficient to induce sustained proinflammatory and anti-tumor effects downstream of IL-12.

Of note, IL-12 levels were monitored at the protein level and did not distinguish between endogenous and transgenic protein. Therefore, hypothetically AAV.IL-12 could have induced the production of endogenous protein. Such a scenario is not supported by our data, however, as IL-12 levels were strictly AAV-dose and tet-dependent and quickly declined within hours – matching the kinetic of reporter gene expression driven by the K19 switch. Finally, AAV-control treated animals or metastasis control groups did not show increased IL-12 levels.

AAV vectors have emerged as the preferred platform for *in vivo* gene therapy, mostly in the context of gene replacement to treat monogenic diseases. However, innate immune recognition of methylated CpG motifs in the transgene cassette induces antigen-specific CD8+ T cell responses against the payload (33). In the case of liver-targeted gene delivery, T-cell-mediated loss of transduced hepatocytes, leading to lack of sustained transgene expression, has been suggested by clinical and pre-clinical data (34–36). We addressed this by removing all CpG sequences from the IL-12 cassette, a strategy that was recently applied in a pre-clinical non-viral IL-12 gene therapy study (37). This optimization did not alter biological potency of the transgene cassette *in vivo* (**Figure 4**). We thereby eliminated one factor potentially confounding clinical dose-response relationship. AAV vector genomes are maintained as episomes in transduced cells, and multi-year transgene expression has been documented in clinical trials, leading to FDA approval of the first liver-directed Hemophilia A gene therapy drug (38). Recent data in non-human primates suggest that persistent transgene expression might be caused by vector genome integration in hepatocytes, rather than by concatemerized episomal genomes (39). This observation calls for a very tight IL-12 regulation, i.e. no detectable expression in absence of tet. Also, along the line of long-term safety, we examined the consequences of low levels of residual IL-12 expression that may occur even in the absence of tet.

Our experiments with the tool construct AAV9.RS-mIL-12 indicated that riboswitch-controlled expression of IL-12 was safer than constitutive expression. A dose of 2.5x10^11^ vg/kg led to premature euthanasia of animals injected with the constitutive control vector AAV9.mIL-12, but not of animals injected with the regulatable construct (**Figures 2C**, **3C**). With the optimized vector AAV8.RS-mIL-12-noCpG, a detailed analysis of the therapeutic window was conducted. Dose titration revealed that doses ≥5x10^10^ vg/kg were highly efficacious, including complete tumor regression, and that 1.5x10^10^ vg/kg still showed considerable tumor growth inhibition (**Figures 5** and **6A**). In general, in individual mice, tumor size reduction was achieved after induction of IL-12 to more than 100 pg/ml in plasma (**Figure 5D**).

To assess safety, we mimicked the treatment protocol of the tumor model in healthy mice and analyzed a wide range of toxicity parameters. Adverse findings depended on the AAV dose, but they were not caused by tet or the vector itself. In a setting with induction of IL-12 by tet, all doses ≤5x10^10^ vg/kg were well tolerated without adverse events (**Figures 7A, B**). Analysis of individual mice over all studies performed here indicated that, in general, IL-12 plasma levels >5000 pg/ml resulted in acute toxicity and early unscheduled termination. In principle, an effective IL-12 plasma level of >100 pg/ml and an acutely toxic level >5000 pg/ml represents a sufficient therapeutic window. By varying the tet dose, the IL-12 level may be adjusted to a safe and efficacious level in each individual. Another study (11) reported 200-fold higher IL-12 levels required for efficacy (>20 ng/ml) and toxicity (>1000 ng/ml) in a mouse model. Interestingly, they also observed a 50-fold window between these thresholds. The deviation in absolute values might be explained by technical differences, such as the quantification method, time-point and matrix (serum/plasma). In most other mouse studies, no clear IL-12 threshold levels for toxicity and efficacy were reported. Maximum tolerated doses observed with recombinant IL-12 protein have only limited relevance for gene therapy, because recombinant IL-12 protein has a short half-life *in vivo*, whereas gene therapy can achieve sustained levels of IL-12. Because of the basal IL-12 expression of AAV8.RS-mIL-12-noCpG in the absence of tet, we analyzed safety without tet induction over 5 weeks (**Figures 7C–N**). No findings were observed at doses ≤1.5x10^10^ vg/kg and minimal findings at 5.0x10^10^ vg/kg, but higher doses resulted in a clear dose-dependent toxicity. Therefore, we defined the range of safe and efficacious AAV doses from 1.5x10^10^ vg/kg with no adverse findings and suboptimal efficacy, to 5.0x10^10^ vg/kg with excellent efficacy, but first hints of potential toxicity. As cancer immunotherapies generally aim at stimulating the immune system, immune-related side effects may not be completely avoidable and are reported for established immunotherapies, such as anti-PD-1 or CAR-T cells (40, 41). Clinically, they are managed, e.g. by anti-IL-6 therapy or general immune suppression with corticosteroids. For AAV.RS-huIL-12, the approved anti-IL-12/IL-23 antibody ustekinumab might be used as a specific inhibitor.

Intratumorally injected IL-12 mRNA promoted Th1 transformation of the tumor microenvironment and anti-tumor immunity in mouse models (42). In a clinical trial in patients with advanced melanoma, local electroporation of an IL-12-encoding plasmid was combined with pembrolizumab. The combination was associated with a higher-than-expected response rate (43). However, non-viral approaches entailing tumor electroporation are best achieved in accessible cancer types, such as melanoma, and are hampered by low IL-12 levels and limited duration of expression.

A protein-based approach aiming at limiting systemic toxicity is the “immunocytokine” NHS-IL-12 that consists of two molecules of IL-12 fused to an antibody that targets tumor necrosis via binding to DNA (44). In a clinical trial with monotherapy, no objective tumor responses were observed (45). However, the immune system was stimulated, and some patients experienced stable disease. It is questionable whether immunocytokines can avoid systemic toxicity, as the cytokine can also interact with immune cells in circulation. Another targeting approach is the fusion of IL-12 to a domain that masks IL-12. The linker used is cleavable by tumor-associated proteases, thus releasing active IL-12 preferentially at the tumor site. Impressive preclinical results have been published, but clinical data is still missing (46, 47).

Liver fibrosis is a common comorbidity of HCC. The altered liver architecture may have a significant impact on the penetration and transduction efficiency of a systemically delivered therapeutic virus. A report on inferior transgene expression in cirrhotic liver achieved by delivery of hepatotropic AAV1 in the portal vein of rats (48) is supported by data we generated. Indeed, in a preclinical mouse model of liver fibrosis we have found that severe fibrosis reduced the transduction efficiency of AAV8 by the order of a magnitude (data not shown). The variability of fibrosis grades between HCC patients complicates the prediction of the clinical dose, but also speaks for a tightly regulatable system to fine-tune IL-12 expression in each patient individually.

More subtle differences in the AAV dose-response PK relationship were observed in healthy mice vs. tumor-bearing mice. In healthy mice, we determined 2.5.x10^10^ vg/kg as the vector dose resulting in no detectable IL-12 levels in plasma without tet administration or 3 days after transient tet exposure (**Figures 2C–E**). In the HCC model though, the same vector dose led to detectable IL-12 levels even without tet administration (**Figure 3B**). While these IL-12 levels were safe, they also showed a treatment benefit in tumor-bearing mice. The IL-12 data confirm the potential to fine-tune expression levels of a therapeutic protein *in vivo* by vector titration and adjusting the ligand-dose in a riboswitch context. Our data show that the K19 riboswitch enables versatile and comparable regulation of different transgenes, including reporters (12), and mouse and human IL-12 cDNA employing different AAV serotypes (this study). The reported lack of function of the K19 aptazyme to switch AAV-mediated transgene expression in the mouse eye (49) is likely partially due to the poor tet exposure in this organ (12).

While our approach represents another part in the growing molecular armory to fight liver cancer, the relatively small therapeutic window that we observed indicates the need for further optimization. Using the K19 aptazyme, we observed dynamic ranges of 5 to 11-fold expression levels in this study which is similar to the 19-fold range achieved when driving reporter gene expression (12). Tet-responsive riboswitches showing comparable dynamic ranges were achieved in C. elegans by leveraging the modularity of combining tet-aptamers with exon-skipping expression platforms (50). A recent report suggests that strongly improved regulation can be achieved in the mouse by using a tet switch design with a poly-A signal in the 5’-UTR and including several aptamers (51).

Achieving controllable gene expression at high dynamic ranges is highly desirable, as evident from various protein-based systems that have been investigated recently, including destabilizing domains and inducible promoters as the most advanced approaches (7, 52). The TET-on system was used to regulate IL-12 in mouse model of CRC (11). This system requires bidirectional expression of both IL-12 and the mutated reverse tet transactivator rtTA under the control of the liver-specific albumin promoter. Doxycycline-induced regulation of IL-12 provided anti-tumor efficacy. However, tolerability was not investigated, and optimization of the inducer dose was not performed. These transcriptional control systems, including the Rheoswitch system, Mifepristone, and classic Tet-ON/OFF promoter systems, suffer from a unifying drawback: They require the expression of DNA-binding proteins that enable transcription after being activated by their cognate ligands (8, 11, 53–55). These DNA-binding proteins represent T-cell epitopes that bear an immunogenic risk. Attempts were undertaken to address this risk for the Tet-promoter control system by engineering versions without certain T cell epitopes for HLA0201 (the most common human HLA serotype). If that is even possible –the protein has to retain specific binding to both the Tet repressor, and to tet –humans with other serotypes may still present epitopes from the resulting protein, given lack of immune tolerance to this exogenous protein (56). These data show that genetic switches with wide dynamic ranges that control transgene expression without additional protein components are required to achieve the full potential of gene therapy technologies.

In conclusion, our data suggest that IL-12 gene therapy can be controlled using an aptazyme approach in a spatio-temporal manner for a safe and efficient immunomodulatory effect using a rational combination of AAV serotype, vector dose, and tet dosing regimen. This approach enabled regulated and repeatable transgene expression in a dose-dependent manner and achieved a defined therapeutic window. As such, the RS-IL-12 approach added safety over non-regulated gene therapy but will require further optimization.

## Data availability statement

The original contributions presented in the study are included in the article/**Supplementary Material**. Further inquiries can be directed to the corresponding authors.

## Ethics statement

Ethical approval was not required for the studies on humans in accordance with the local legislation and institutional requirements because only commercially available established cell lines were used. The animal study was approved by Regierungspräsidium Tübingen, Germany, or Regierungspräsidium Freiburg, Germany. The study was conducted in accordance with the local legislation and institutional requirements.

## Author contributions

MD: Conceptualization, Investigation, Visualization, Writing – original draft. RK: Conceptualization, Investigation, Methodology, Project administration, Supervision, Writing – review & editing. PV-G: Investigation, Writing – review & editing. BS: Conceptualization, Methodology, Resources, Writing – review & editing. TS: Investigation, Writing – review & editing. PG: Investigation, Writing – review & editing. GA: Formal Analysis, Methodology, Software, Visualization, Writing – original draft. AG: Investigation, Writing – review & editing. IL: Investigation, Writing – review & editing. HK: Methodology, Software, Writing – review & editing. JP: Conceptualization, Writing – review & editing. SK: Conceptualization, Writing – review & editing. MK: Conceptualization, Project administration, Supervision, Writing – original draft, Writing – review & editing. FI: Conceptualization, Project administration, Supervision, Writing – original draft, Writing – review & editing. SM: Investigation, Writing – review & editing. SH: Investigation, Writing – review & editing.
