# Supplementary figures and images for: Riboswitch-controlled IL-12 gene therapy reduces hepatocellular cancer in mice
Source: Front Immunol. 2024 Mar 15;15:1360063. doi: 10.3389/fimmu.2024.1360063 (PMC10979303; doi:10.3389/fimmu.2024.1360063)

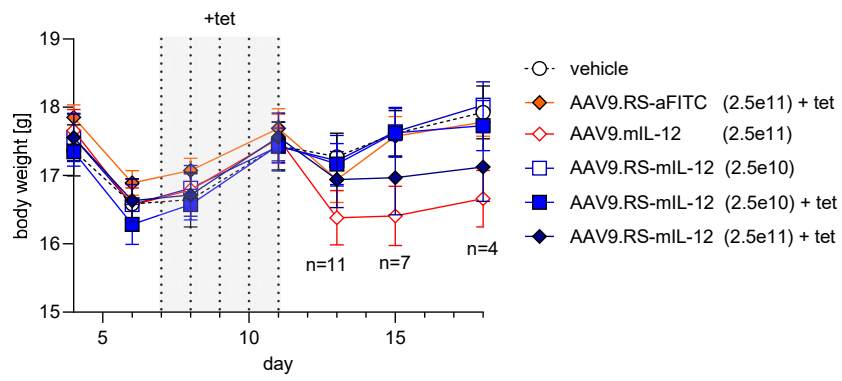

# **SUPPLEMENTARY FIGURE 1**

Body weight development after AAV application and tet treatment.

Supplement: Supplementary file 1 [file Image_1.pdf]

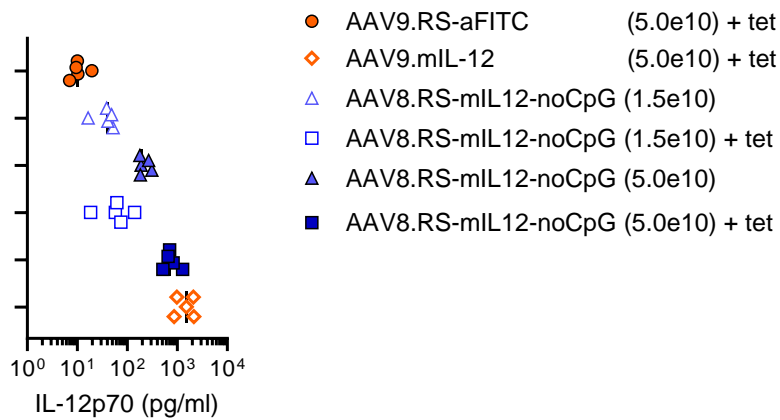

### SUPPLEMENTARY FIGURE 3

mIL-12 levels in plasma collected on day 8 (6h after tet application).

Supplement: Supplementary file 3 [file Image_3.pdf]
