# Supplementary material for: Riboswitch-controlled IL-12 gene therapy reduces hepatocellular cancer in mice
Source: Front Immunol. 2024 Mar 15;15:1360063. doi: 10.3389/fimmu.2024.1360063 (PMC10979303; doi:10.3389/fimmu.2024.1360063)
Supplement: Supplementary file 2 [file Image_2.pdf]

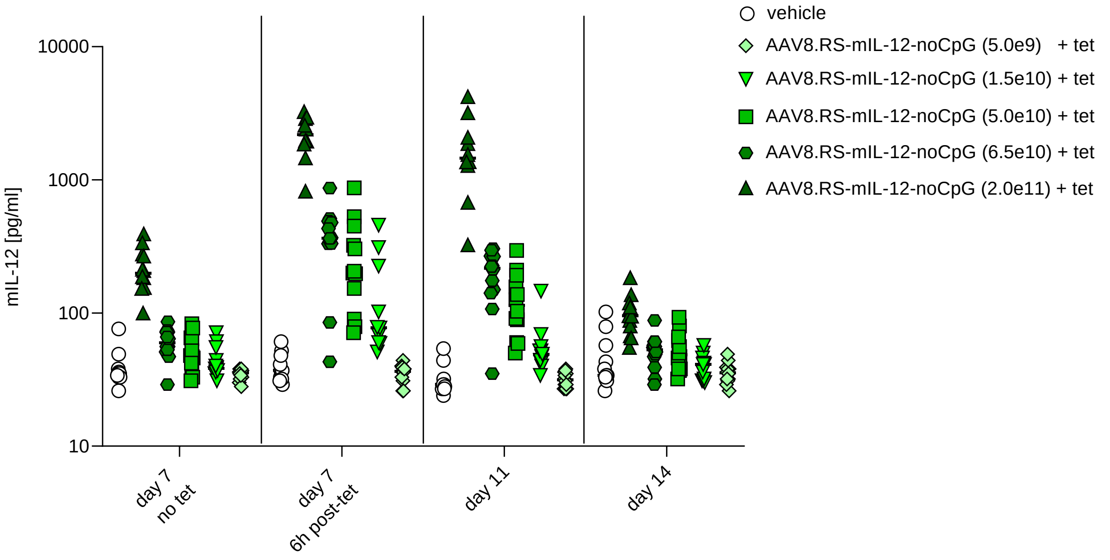

SUPPLEMENTARY FIGURE 2

mIL-12 level in plasma on day 7 (before and 6h after tet activation), on day 11 (6h after tet activation), and on day 14 (3 days after last tet application).
