# Supplementary material for: Riboswitch-controlled IL-12 gene therapy reduces hepatocellular cancer in mice
Source: Front Immunol. 2024 Mar 15;15:1360063. doi: 10.3389/fimmu.2024.1360063 (PMC10979303; doi:10.3389/fimmu.2024.1360063)
Supplement: Supplementary file 4 [file Image_4.pdf]

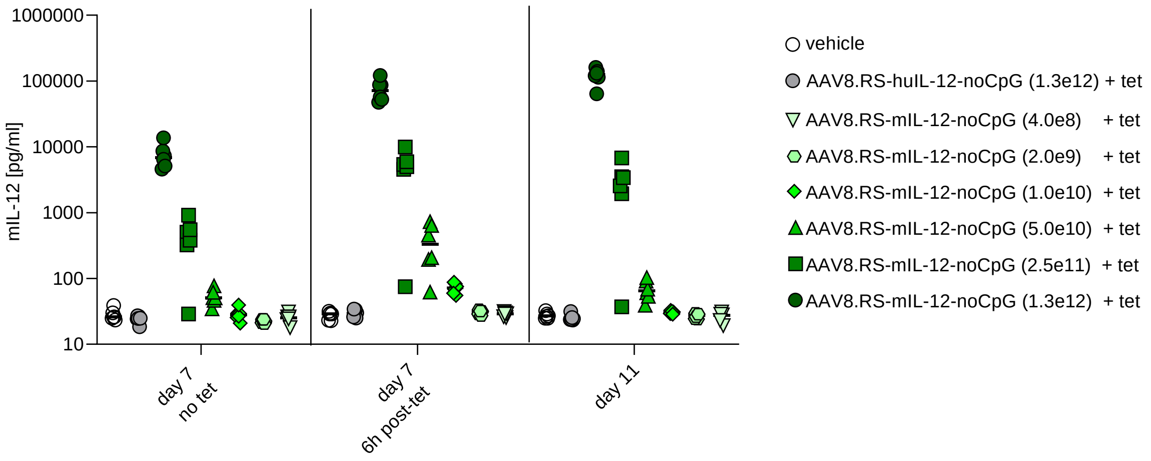

SUPPLEMENTARY FIGURE 4

mIL-12 levels in plasma collected on day 7 (before and 6h after tet application) and day 11 (6h after tet application).
